# Validity and Reliability of Japanese-Language Self-reported Measures for Assessing Adults Domain-Specific Sedentary Time

**DOI:** 10.2188/jea.JE20170002

**Published:** 2018-03-05

**Authors:** Kaori Ishii, Ai Shibata, Satoshi Kurita, Shohei Yano, Shigeru Inoue, Takemi Sugiyama, Neville Owen, Koichiro Oka

**Affiliations:** 1 Faculty of Sport Sciences, Waseda University, Saitama, Japan; 2 Faculty of Health and Sport Sciences, University of Tsukuba, Tokyo, Japan; 3 Graduate School of Sport Sciences, Waseda University, Saitama, Japan; 4 Department of Preventive Medicine and Public Health, Tokyo Medical University, Tokyo, Japan; 5 Institute for Health & Ageing, Australian Catholic University, Melbourne, Australia; 6 Swinburne University of Technology, Melbourne, Australia; 7 Behavioural Epidemiology Laboratory, Baker IDI Heart and Diabetes Institute, Melbourne, Australia

**Keywords:** domain-specific sedentary behavior, questionnaire, accelerometer, measurement, population

## Abstract

**Background:**

Good quality measures of Japanese adults’ sedentary behaviors are needed to accurately assess correlates of specific sedentary behaviors. The present study assessed criterion validity of total sedentary behavior and test-retest reliability of six domain-specific sedentary behaviors.

**Methods:**

We administered a questionnaire, based on previous studies, that measured domain-specific sedentary behaviors. To examine validity, agreement between self-reported time spent in sedentary behaviors from the questionnaire and objectively-measured sedentary time using accelerometers was compared among 392 adults (aged 40–64 years) in two Japanese cities. For reliability, a 2-week interval test-retest was administered to a convenience sample of 34 participants.

**Results:**

The correlation between total self-reported and objectively measured sedentary time was significant (all *P* < 0.001) and fair-to-good for workdays (ρ = 0.57) and whole week (ρ = 0.49), but was low for non-workdays (ρ = 0.23). The difference between the two measures was significant for whole week (*z* = −2.25, *P* = 0.03) and non-workdays (*z* = −5.50, *P* < 0.001), but was not significant for workdays (*z* = −0.60, *P* = 0.55). There was a significant positive association between the difference in the two measures and the average of these two measures (workdays: *r* = 0.53; non-workdays: *r* = 0.45; and whole week: *r* = 0.54, all *P* < 0.001). There was fair-to-good test-retest reliability of total sedentary time for each domain (workdays: interclass correlation coefficient [ICC] = 0.77, non-workdays: ICC = 0.53, and whole week: ICC = 0.7; all *P* < 0.01).

**Conclusions:**

The scale of domain-specific sedentary behaviors is reliable for estimating where and for what purpose Japanese adults spend their sedentary time, and total sedentary time is valid for workdays and the whole week.

## INTRODUCTION

Recent studies have reported that time spent in sedentary behaviors, such as leisure-time sitting and sitting at work, is associated with an increased risk of type 2 diabetes and cardiovascular disease,^1^ after controlling for physical activity levels.^2^ However, a large proportion of the population in developed countries spend the majority of their waking hours sedentary.^3^ A previous descriptive epidemiological study of participants aged 18–65 years from 20 countries reported that the median sitting time was 300 min/day.^4^ Thus, reducing sedentary time, in addition to increasing physical activity, is an important strategy to enhance population health.

In order to conduct high-quality epidemiological and behavioral studies and to monitor population prevalence and variations, it is important to identify sedentary time accurately. Recently, studies have been increasingly using device-based measures, such as accelerometry, to assess sedentary time. However, such objective measures do not provide domain-specific information about the setting (where) and purpose (what) of the behavior. The fact is that sedentary behavior occurs in a variety of contexts. Identifying the characteristics of domain-specific sedentary behavior may help when designing more effective intervention strategies for reducing sedentary time and help to identify the domains on which it will be most important to focus. Moreover, it is not always feasible to collect objective measures in large-scale surveillance or time-sensitive examinations, as these can induce strain in participants and can result in cost-based and logistic difficulties related to device use. In contrast, self-report questionnaires can elicit information about the behavioral context, can be easily administered, and are even low in cost for large-scale epidemiologic studies.^5^

Although a few studies have assessed domain-specific sedentary time, these studies have been limited to elderly people,^6^ university students/faculty,^7^^,^^8^ adolescents,^5^ and workers.^9^ Furthermore, the number of participants used to assess the validity of measures using an accelerometer in such studies has been low (range; spearman *r* = 0.14–0.46,^5^^,^^6^^,^^9^ interclass correlation coefficient [ICC] = 0.20–0.64^7^^,^^8^). There are domain-specific sedentary behavior measures produced for adults.^10^^,^^11^ However, one assesses sitting in the past day^11^ and showed low test-retest correlations, suggesting day-to-day variability of sitting time. The other measure asked sitting time for “each day” without a clear time frame for assessment.^10^ Gold standard international questionnaires used to measure physical activity and sedentary behavior, such as the International Physical Activity Questionnaire^12^ and Global Physical Activity Questionnaire,^13^ were administered to assess the estimated activity in a typical week or the last 7 days for physical activities that people usually perform as part of their everyday lives. When assessing usual sedentary behavior, any single day has the potential to not follow the usual pattern. Therefore, the assessment of the last 7 days is necessary to estimate usual sedentary behavior without focusing on a specific day of the week. Both domain-specific sedentary behavior^10^^,^^11^ measures asked sitting in the following five domains: work, transport, television, computer use, and other leisure time (an additional two domains of reading and hobby were assessed in Clark et al’s study^11^). Given that transport can be done either by car or public transport, which are likely to have differential impacts on health, these two may have to be measured separately. The purpose of the present study is to assess the criterion validity of total sedentary time and test-retest reliability of a questionnaire that measures time spent sitting in the last 7 days in the following six domains: (1) driving or riding in a car; (2) using public transport; (3) at work; (4) watching television, videos, and DVDs; (5) using a computer, cell phone, and tablet personal computer (PC) outside of working hours; and (6) in leisure time (eg, reading, not including watching television, videos, and DVDs).

## METHODS

### Participants

The assessment of criterion validity was conducted from July to December 2013 and April 2014 to April 2015. A total of 6,000 potential residents aged 40–64 years living in two Japanese localities, Koto Ward and Matsuyama City, were randomly selected from the residential registries of their respective cities. Koto (population: 480,271 in January 2013) is one of the 23 wards within the Tokyo Metropolitan area, and Matsuyama (population: 517,838 in June 2014) is a mid-sized regional city in southwest Japan. Koto Ward and Matsuyama City represent high-density and low-density urban regions, respectively. Potential participants were stratified by gender (men/women) and age bracket (40–44 years, 45–49 years, 50–54 years, 55–59 years, and 60–64 years). First, invitation letters explaining the study were sent to all potential participants. To encourage a response, potential participants were told that a 500-yen book voucher would be offered to those who returned the questionnaire. Non-respondents were sent one reminder about the questionnaire. A total of 864 individuals (14.4% overall response rate; 437 responders [14.6%] from Koto and 427 responders [14.2%] from Matsuyama) replied to the invitation. Then, self-administered questionnaires, which included questions about sociodemographic variables, sedentary behaviors, height, and weight, were mailed to those who responded, along with an accelerometer (Active style Pro HJA-350IT; Omron Healthcare, Kyoto, Japan). A total of 778 completed both the questionnaire and the accelerometer measurement (90.0% overall response rate; 85.8% from Koto and 94.4% from Matsuyama). For reliability, a 2-week interval test-retest was conducted with a convenience sample of 36 adult volunteers who were recruited via word of mouth through personal connections. Data from 392 adults involved in the assessment of validity and 34 adults involved in the assessment of reliability, who fully completed both the questionnaire and the accelerometer measurement, were included in the analysis.

### Standard protocol approvals, registrations, and patient consent

All participants signed an informed consent before answering the questionnaire. The Ethics Committee of Waseda University, Japan, approved the study prior to its commencement (2012-269, 2013-264). The present study was conducted in accordance with the principles articulated in the Declaration of Helsinki of 2013.

### Measures

#### Self-reported sedentary behavior

A scale consisting of six items was administered with a 1-week recall period. Participants were asked to report daily average sedentary time (hours and minutes) over the past 7 days, separately for workdays (weekday for non-employed) and non-workdays (weekend for non-employed) across the following six domains: while (1) being transported to and from a place by car; (2) using public transport; (3) at work; (4) watching television, videos, and DVDs; (5) using a computer, cell phone, or tablet PC outside of working hours; and (6) in leisure time (excluding watching television, videos, and DVDs). The scale was newly developed with reference to previous studies.^6^^,^^7^^,^^10^^,^^11^ Main changes included separation of car use and public transport use (as discussed above) and incorporation of recent technologies that could involve sitting. Unemployed individuals were instructed to consider workdays as weekdays and non-workdays as weekend days. Total minutes of daily average sedentary time were calculated by summing all six items separately for workdays and non-workdays. Total minutes of daily average sedentary time for a whole week was then calculated as [total time of workday × working days per week + total time of non-workday × (7-working days per week)]/7. Reliability was assessed by a 2-week test-retest protocol. Participants self-reported their sitting time twice within the 2-week period.

#### Objective measurement of sedentary time

The objectively measured sedentary time was evaluated for 7 consecutive days using a validated tri-axial accelerometer (Active style Pro, HJA-350IT; Omron Healthcare, Kyoto, Japan).^14^^,^^15^ The data were collected in 1-min epochs and expressed as metabolic equivalents (METs). Participants were instructed to wear the accelerometer throughout the day, except during sleep and water-related activities (eg, bathing or swimming) or while participating in activities, such as contact sports (eg, soccer or rugby). Non-wear time was defined as intervals of at least 60 consecutive min of 0 METs, with allowance for up to 2 min of observations of some limited movement (<1.0 METs) within these periods, and it was regarded as valid when the device was worn for at least 10 hours per day. Participants were included for analysis if they had complete data for a minimum of 4 days, including at least 1 non-workday. Sedentary behavior was defined as any activity in which accelerometer-estimated intensity was ≤1.5 METs.^16^ Total daily minutes of sedentary time was calculated for the week and separately for workdays and non-workdays.

#### Sociodemographic factors

Participants provided information about their sociodemographic attributes, such as gender, age, height, weight, and usual number of working days per week. Additionally, participants involved in the validity assessment were asked to provide information about their educational level (graduate school or university, 2 years of university education or equivalent, college, high school, or junior high school), employment status (full time, part time, full-time home-worker, student, or unemployed), marital status (married or unmarried), living arrangements (cohabitating or living alone), and household income level per year (<3, ≥3–<5, ≥5–<7, ≥7–<10, or ≥10 million yen).

### Statistical analyses

#### Validity

Spearman’s rho was used to determine the correlation between total self-reported sedentary behaviors and objectively measured sedentary time for workdays, non-workdays, and the whole week. Total self-reported sedentary behaviors was compared with the median objectively measured sedentary time for workdays, non-workdays, and the whole week using Wilcoxon tests. Bland-Altman plots^17^ were used to examine the differences between self-reported sedentary behaviors and objectively-measured sedentary time, and the average of the two measures. Plots with the mean difference and limits of agreement (plus or minus 1.96 standard deviations) are reported. Pearson’s correlation was used to assess the correlations between the mean difference (self-reported time − objectively measured time) across the average values of self-reported and objectively measured sedentary time ([self-reported + objectively measured]/2) for workdays, non-workdays, and the whole week. The strength of correlation as indicated by Spearman’s rho was interpreted as weak (<0.30), low (≥0.30 to <0.50), moderate (≥0.50 to <0.70), strong (≥0.70 to <0.90), and very strong (≥0.90),^18^ and Pearson’s correlation was interpreted as insubstantial (<0.1), small (≥0.1 to <0.3), moderate (≥0.3 to <0.50), and large (≥0.50).^19^ All statistical analyses were performed with SPSS 22.0J for Windows (SPSS Inc., Chicago, IL, USA). A *P* value <0.05 was considered statistically significant.

#### Reliability

ICCs with 95% confidence intervals were used to assess test-retest reliability by comparing participants’ responses on the self-reported domain-specific items and total sedentary behaviors at Time 1 and Time 2 for workdays, non-workdays, and the whole week. The ICCs were calculated using a two-way mixed model based on absolute agreement. The ICC was interpreted as indicating poor reliability (<0.4), fair to good reliability (≥0.4 to <0.75), and excellent reliability (≥0.75).^20^

## RESULTS

### Participant characteristics

The participant characteristics are shown in Table 1. Among the participants involved in the reliability and validity assessment, fewer men were included in the validity assessment than in the reliability assessment, and participants in these groups had a mean age of 50.1 years and 40.3 years, respectively. Of these, more than half had attained a high level of education and had a high household income, and over 80% were employed, married, and/or lived with other people. Both the reliability and validity participants were mostly normal (<25.0 kg/m^2^) in their body mass index (BMI) (76.5% and 82.1%, respectively). Included participants were younger (*t* = 5.15, *P* < 0.001), had lower BMI (*t* = 1.98, *P* = 0.05), had higher levels of education (*x*^2^ = 29.5, *P* < 0.001), and had higher levels of household income (*x*^2^ = 13.0, *P* = 0.01).

**Table 1.** Characteristics of participants

	Participants for the validity assessment		Participants for the reliability assessment	
	*n*	%	*N*	%
Total	392	100	34	100
Sex				
Men	156	39.8	20	58.8
Women	236	60.2	14	41.2
Age, years				
Mean (SD)	50.1	(7)	40.3	(11.4)
Educational level				
Graduate school or university	186	47.4		
2 years of university education or equivalent college	85	21.7		
High school	116	29.6		
Junior high school	5	1.3		
Employment status				
Full time	234	59.7		
Part time	84	21.4		
Full-time Home-worker	18	4.6		
Student	55	14.0		
Unemployed	1	0.3		
Marital status				
Married	322	82.1		
Unmarried	70	17.9		
Living condition				
Living with others	356	90.8		
Living alone	36	9.2		
Household income level				
<3,000,000 yen	55	14.0		
3,000,000 to <5,000,000 yen	106	27.0		
5,000,000 to <7,000,000 yen	70	17.9		
7,000,000 to <10,000,000 yen	91	23.2		
≥10,000,000 yen	70	17.9		
Body mass index				
Mean (SD)	22.2	(3.4)	22.5	(3.1)

SD, standard deviation.

### Validity

**Figure 1.**
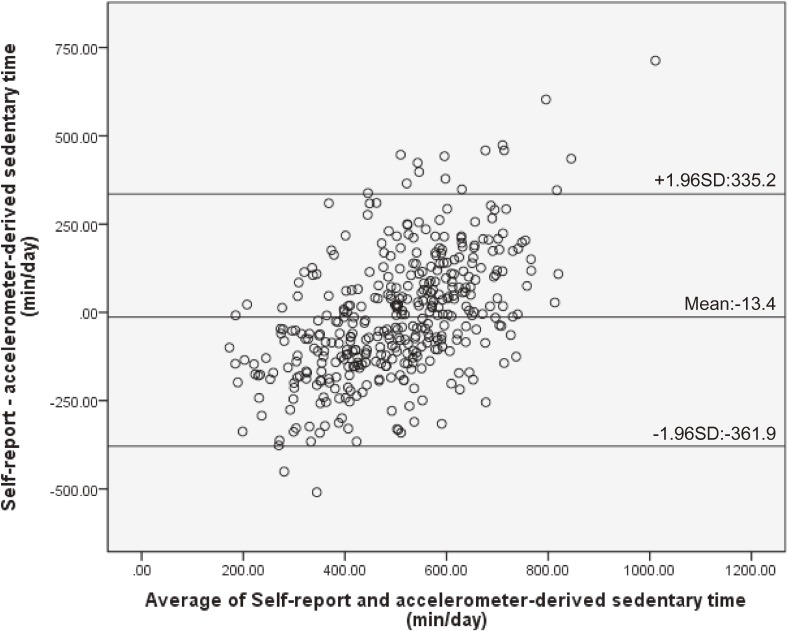
Bland-Altman plot of the total self-reported sedentary behaviors with objectively measured sedentary time for the whole week. Mean differences and limits of agreement (plus or minus 1.96 standard deviations) are shown in the figure. SD, standard deviation.

**Figure 2.**
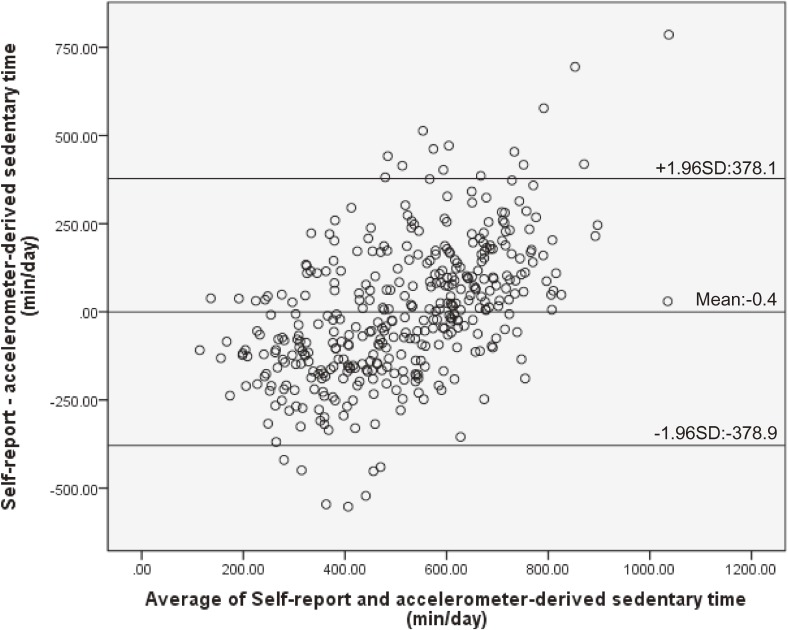
Bland-Altman plot of the total self-reported sedentary behaviors with objectively measured sedentary time for the workdays. Mean differences and limits of agreement (plus or minus 1.96 standard deviations) are shown in the figure. SD, standard deviation.

**Figure 3.**
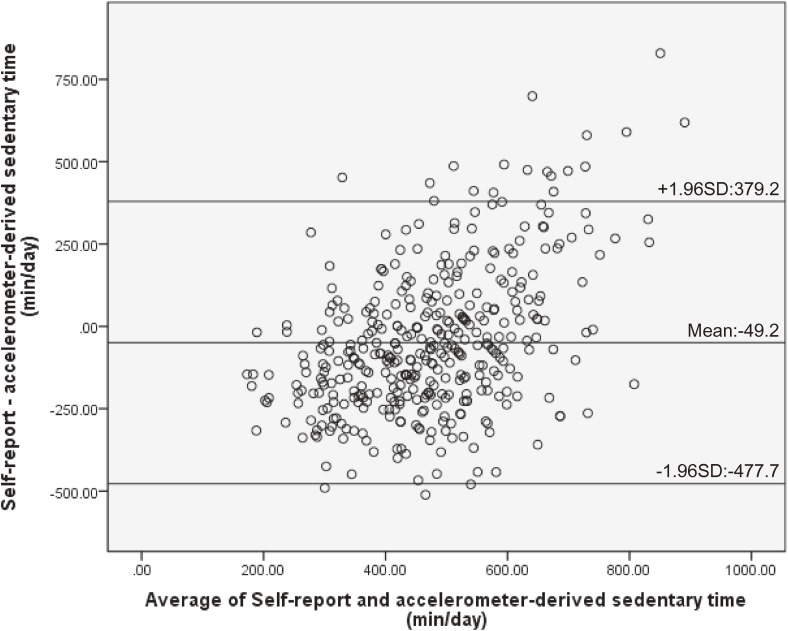
Bland-Altman plot of the total self-reported sedentary behaviors with objectively measured sedentary time for the non-workdays. Mean differences and limits of agreement (plus or minus 1.96 standard deviations) are shown in the figure. SD, standard deviation.

The rank-order correlation between total self-reported sedentary behaviors and objectively measured sedentary time was significant and low to moderate for workdays (ρ = 0.57, *P* < 0.001) and whole week (ρ = 0.49, *P* < 0.001); however, it was weak for non-workdays (ρ = 0.23, *P* < 0.001). The differences between self-reported sedentary behaviors and objectively measured sedentary time were significant for the whole week (*z* = −2.25, *P* = 0.03) and non-workdays (*z* = −5.50, *P* < 0.001), but it was not significant for workdays (*z* = −0.60, *P* = 0.55; Table 2). The Bland-Altman plots showing the agreement between self-reported sedentary behaviors and objectively measured sedentary time are presented in Figure 1, Figure 2, and Figure 3. There was a significant positive correlation between the difference of the two measures and the average of them (workdays: *r* = 0.53, *P* < 0.001; non-workdays: *r* = 0.45, *P* < 0.001; whole week: *r* = 0.54, *P* < 0.001). The limits of agreement (plus or minus 1.96 standard deviations) were −378.9 to 378.1 min for workdays, −477.7 to 379.2 min for non-workdays, and −361.9 to 335.2 min for the whole week.

**Table 2.** Differences and correlation between total self-reported sedentary behaviors and median objectively measured sedentary time

	*z*	*P*	Objectively measured sedentary time (minutes)		Self-reported sedentary behaviors (minutes)		Correlation between self-reported sedentary behaviors and objectively measured sedentary time	

			Median	25%–75% tile	Median	25%–75% tile	ρ	*P*
Workday	−0.60	0.55	524.7	411.8–614.6	510.9	330.0–684.7	0.57	<0.001
Non-workday	−5.50	<0.001	498.4	410.8–578.7	422.2	301.3–554.0	0.23	<0.001
Whole week	−2.25	0.03	512.1	429.8–589.0	506.7	350.7–636.4	0.49	<0.001

### Test-retest reliability

Table 3 presents the test-retest reliability of each domain-specific sedentary behavior and total sedentary behavior. There was fair to good test-retest reliability between Time 1 and Time 2 for the total sedentary behaviors calculated from each domain’s sedentary behaviors during workdays (ICC = 0.77, *P* < 0.01), non-workdays (ICC = 0.53, *P* < 0.01), and the whole week (ICC = 0.74, *P* < 0.01). For each specific domain, the workdays and whole week had fair to good reliability (ICCs ranging from 0.45–0.89, all *P* < 0.001); however, the reliability was considerably lower and not significant for work (ICC = −0.07, *P* = 0.65) and public transport use (ICC = 0.20, *P* = 0.13) on non-workdays.

**Table 3.** Test-retest reliability of self-reported sedentary behavior

Sedentary domain	ICC	95% CI		*P*	Minutes at Time 1		Minutes at Time 2	
					Median	25%–75% tile	Median	25%–75% tile
Workday								
Car	0.85	0.71	0.92	<0.001	7.5	0.0–25.7	4.0	0.0–15.0
Public transport	0.60	0.33	0.78	<0.001	7.5	0.3–41.7	4.7	0.0–54.0
Work	0.89	0.80	0.95	<0.001	112.5	55.7–240.0	110.0	61.4–255.0
Television	0.76	0.58	0.88	<0.001	90.0	27.1–180.0	93.3	41.3–162.0
Computer use	0.72	0.51	0.85	<0.001	51.7	22.5–90.0	63.0	25.0–116.7
Leisure	0.45	0.15	0.68	<0.001	45.0	25.0–65.0	60.0	37.8–85.5
Total	0.77	0.60	0.88	<0.001	370.0	285.7–600.0	426.0	315.0–570.0
Non-workday								
Car	0.53	0.24	0.74	<0.001	14.5	0.0–51.4	8.5	0.0–50.0
Public transport	0.20	−0.15	0.50	0.13	3.8	0.0–37.5	8.0	0.0–36.0
Work	−0.07	−0.40	0.28	0.65	3.6	0.0–34.5	7.7	0.0–40.6
TV	0.79	0.63	0.89	<0.001	173.3	105.0–256.7	180.0	102.0–264.0
Computer use	0.72	0.51	0.85	<0.001	100.0	51.7–175.0	100.0	37.5–167.1
Leisure	0.46	0.14	0.69	<0.001	90.0	46.2–150.0	128.0	74.1–170.0
Total	0.53	0.24	0.73	<0.001	461.5	330.0–640.0	491.7	422.0–590.0
Whole week								
Car	0.83	0.69	0.91	<0.001	4.3	0.0–37.1	3.2	0.0–34.3
Public transport	0.47	0.17	0.70	<0.001	11.8	0.3–41.4	10.0	0.8–55.7
Work	0.83	0.69	0.91	<0.001	78.9	41.0–205.7	68.6	41.1–205.7
TV	0.82	0.67	0.91	<0.001	107.1	60.0–188.6	120.0	60.0–188.6
Computer use	0.74	0.54	0.86	<0.001	65.7	33.4–131.4	70.7	38.6–130.7
Leisure	0.53	0.25	0.73	<0.001	62.9	32.1–94.3	79.3	61.7–115.7
Total	0.74	0.55	0.86	<0.001	394.3	308.6–587.1	455.7	375.0–591.4

CI, confidence interval; ICC, interclass correlation coefficients.

## DISCUSSION

The present study assessed the criterion validity of total sedentary time and test-retest reliability of six domain-specific items that assessed sedentary time among randomly selected community-dwelling adults. The test-retest reliability was fair-to-good for most domain-specific sedentary behavior and total sedentary behaviors, except for sitting during public transport use and at work on non-workdays. The findings suggested that the present scale had acceptable validity when compared to objectively measured sedentary time; however, validity was poor, as suggested by the slightly wide limits of agreement in ranking participants in terms of sedentary behavior in comparison to the limits reported in previous studies, as it underestimated the actual duration of sedentary behavior (in particular, on non-work days). Therefore, the scale may be more suitable for use in large-scale studies than in studies assessing individual’s sedentary time. Most studies^5^^–^^11^ of validity and reliability related to domain-specific sedentary behavior have used a limited set of participants, such as only women or a university population, and they were conducted in Europe or Australia. Therefore, it is meaningful that the present study developed the scale for the first time in Asia and that it was conducted with randomly selected community-dwelling adults.

The validity correlations of the scale for total sedentary time were acceptable for workdays and the whole week. Compared with the findings of previous studies of the total of multiple-domain sedentary behaviors and objectively measured sedentary time (ρ = 0.30,^6^ ρ = 0.32,^21^ and ρ = 0.15^22^), the present result was higher (ρ = 0.57). Moreover, the difference between self-reported sedentary behaviors and objectively measured sedentary time during workdays was small (median difference: −13.8 min/day) and not statistically significant. However, the self-reported scale significantly underestimated sedentary time during non-workdays (median difference: −76.2 min). These results compare well with a previous study.^7^ Clemes and colleagues^7^ reported that the mean difference between objectively measured sedentary time and total sitting time as assessed from a domain-specific questionnaire was −13.7 min during weekdays (*P* > 0.05). The present results suggest that the scale examined in this study can be useful in assessing the ranking and amount of total sedentary behaviors for workdays and for the whole week.

Although differences in the median measures were observed, the Bland-Altman limits of agreement between the total sedentary time and objectively measured sedentary time were in the similar range for workdays, non-workdays, and for the whole week (workday: ±378.5 min; non-workday: ±428.5 min; whole week: ±348.5 min) when compared with previous studies (weekdays: ±368.3 min, weekend: ±574.3 min^7^; weekdays: ±277.2 min, weekends: ±328.8 min, all days: ±259.2 min^23^). However, the limits of agreement indicated that differences existed between measures for some individuals. Moreover, a significant positive association was found between the difference of the two measures and the average of these two measures, which indicated that the amount of overestimate via the self-report measure would be greater as overall sedentary time gets longer. The reason for this result may be that, when responding about domain-specific sedentary behaviors, the participants may not be reporting one domain, such as when using a tablet PC while using public transport to and from a place, especially during non-workdays. Participants were more likely to show the pattern of behavior of combining domains during non-workdays than workdays. However, it is not clear why longer total sedentary behavior was positively associated with greater overestimation. Further research is needed to fully understand the relationships. Therefore, when using the scale, it is necessary to pay attention to the fact that a long total sedentary time was associated with overestimated self-reported sedentary time.

Acceptable reliability was shown for total sedentary behaviors. The reliability in the present study (ICC = 0.74) was higher than in previous studies of total sedentary time calculated from domain-specific sedentary time among older adults (ICC = 0.52) with a 1-week recall^6^ and adults (ICC = 0.50) with recall over the past day.^11^ However, reliability was low for the domain-specific sedentary behavior during public transport use and work on non-workdays. Moreover, the reliability of other domain-specific sedentary behavior during non-workdays was slightly lower than for workdays and for the whole week. Marshall and colleagues^10^ and Clark and colleagues^24^ suggested that the assessment of workday sedentary time is more reliable than that for non-work days because workday (weekday) behaviors are routine whereas non-workday (weekend) behaviors can vary from week to week. A longer time frame (eg, the preceding month) may be necessary to assess non-work day sedentary behaviors reliably.

The validity and reliability were acceptable for the total sedentary behaviors as a sum of multiple domains. For people who engaged in sedentary behavior, the domains were different during workdays and non-workdays. Because sedentary behavior is likely to occur across all working hours, it may be difficult to capture total sedentary behavior with a single item. Therefore, as the present scale assesses not only domain-specific sedentary behaviors but also total sedentary time, it is an acceptably valid and reliable scale that is useful for evaluating sedentary time. The scale could be useful when information is needed about which domain-specific sedentary behavior is performed (eg, which domain should be targeted in order to decrease sedentary behavior).

### Limitations

The present study has some limitations to consider while interpreting the results. First, the study respondents for validity were slightly different from the general population. However, the present study population may be considered to share the characteristics of the general population because the present study randomly selected participants from a registry of residential addresses of each city, which allowed an equal number of responses to be obtained from both genders and from each age group category between 40 and 64 years. Second, as the study included only participants aged 40–64 years, the generalizability of the present findings to other age groups is unclear and requires further assessment. Finally, the present study was unable to examine the validity of each domain-specific sedentary behavior; further studies, in which specific sedentary behaviors are examined objectively, are required to assess the validity of sedentary behavior in each domain.

In spite of these limitations, no other study has been conducted on this topic in a randomly recruited group of Japanese adult participants; thus, the findings from the present study contribute to a greater understanding of domain-specific sedentary behavior and may help to develop strategies to promote public health and well-being through decreasing sedentary behavior in Japan.

### Conclusion

The present study shows that this scale to assess domain-specific sedentary behaviors has acceptable validity and reliability for workdays and the whole week in a randomly selected Japanese population. However, the validity and reliability were relatively lower for non-workdays than for workdays and the whole week. The scale is appropriate for estimating total sedentary time (as the sum of specific sedentary behaviors) in a large-scale surveillance or time-sensitive examination, in which it is not always feasible to use objective measures because of the cost of administering the instrument or the length of the period of use.
